# Anti-Platelet Therapy with Clopidogrel Prevents Endothelial Dysfunction and Vascular Remodeling in Aortas from Hypertensive Rats

**DOI:** 10.1371/journal.pone.0091890

**Published:** 2014-03-17

**Authors:** Fernanda R. Giachini, Romulo Leite, David A. Osmond, Victor V. Lima, Edward W. Inscho, R. Clinton Webb, Rita C. Tostes

**Affiliations:** 1 Institute of Biological Sciences and Health, Federal University of Mato Grosso, Barra do Garças, MT, Brazil; 2 School of Pharmacy, Federal University of Ouro Preto, Ouro Preto, MG, Brazil; 3 Department of Physiology, Georgia Regents University, Augusta, Georgia, United States of America; 4 Department of Pharmacology, Ribeirao Preto Medical School, University of Sao Paulo, Ribeirao Preto, SP, Brazil; University of Southampton, United Kingdom

## Abstract

The aim was to investigate the beneficial effects of clopidogrel in thoracic aorta function and structure and to characterize if P2Y_12_ receptors contribute to these effects. Male Sprague Dawley rats were infused with angiotensin II [(Ang II) 60 ng.min^−1^, 14 days] or saline (control rats) and were simultaneously treated with clopidogrel (10 mg.kg^−1^.day^−1^) or vehicle. After 14 days, systolic blood pressure (mmHg) was similar in Ang II-hypertensive rats treated with clopidogrel or vehicle (199±9 vs. 190±11, respectively). Systolic blood pressure in control rats was not altered by clopidogrel treatment (128±1 vs. vehicle, 134±2). Endothelium-dependent relaxation induced by 2-MeS-ADP was decreased in aortas from vehicle-treated Ang II-hypertensive rats, compared to vehicle-treated control rats. This response was elicited via activation of P2Y_1_ and P2Y_12_ receptors. In the presence of L-NAME and indomethacin, 2-MeS-ADP induced contraction and this response was augmented in vehicle-treated Ang II-hypertensive rats, compared to vehicle-treated control rats. The contraction to 2-MeS-ADP was evoked by P2Y_13_ and P2Y_12_ receptor activation. Clopidogrel-treatment did not normalize relaxation or contractile responses induced by 2-MeS-ADP in aortas from Ang II-hypertensive rats. P2Y_1_ and P2Y_12_ protein expression was increased, whereas P2Y_13_ receptor expression was reduced in aorta from vehicle-treated Ang II-hypertensive rats. Endothelium-dependent relaxation upon acetylcholine-stimulation was reduced in vehicle-treated Ang II-hypertensive rats, and clopidogrel treatment was effective in improving endothelial function. Clopidogrel also prevented vascular remodeling, evidenced by augmented media thickness in aortas from Ang II-hypertensive rats. Clopidogrel has beneficial effects on the aortic endothelium of Ang II-hypertensive rats, but its effects do not seem to be directly related to the presence of P2Y_12_ receptors in this vessel.

## Introduction

Extracellular nucleotides are released from several sources, including sympathetic nerves, platelets, endothelial and inflammatory cells. Adenosine-5′-diphosphate (ADP) is stored in high concentration in platelets and this nucleotide is released in response to platelet activation [1]. It is well known that ADP is a pro-aggregating agent [2], [3] and also activates platelet function during arterial thrombosis [4]. ADP elicits platelet aggregation through the activation of the P2Y_1_ and P2Y_12_ receptors expressed by platelets [5]. ADP has been shown to elicit vasorelaxation, as well as to cause vasoconstriction, through the activation of purinoceptors in the vascular endothelium and smooth muscle cells, respectively [6].

Clopidogrel is a pro-drug converted in the liver into an irreversible P2Y_12_ receptor antagonist [5]. Clopidogrel alone or associated with aspirin has been used in the treatment and prevention of cardiovascular diseases [7], [8]. In addition to its inhibitory effects on platelet function, clopidogrel has anti-inflammatory properties [9], [10]. Hence, a reduction of platelet-leukocyte interaction and P-selectin expression has been described, whereas these effects could not be demonstrated with aspirin [11], [12], [13].

More recently, the effects of clopidogrel on vascular function have been evaluated. Clopidogrel improves endothelial function in patients with stable coronary artery disease [14]. In addition, clopidogrel treatment normalizes augmented phenylephrine (PE)-induced vascular contraction and restores impaired relaxation to acetylcholine (ACh) in mesenteric arteries from hypertensive rats [15]. However, it is not clear if this effect is restricted to mesenteric arteries, or whether beneficial effects of clopidogrel treatment are extended to other vascular beds, nor if clopidogrel prevents structural and functional changes of the affected vasculature.

Elevated systemic blood pressure results in high intravascular pressure, but the main complications related to hypertension, which include coronary heart disease, ischemic strokes and peripheral vascular disease, are related to thrombosis rather than hemorrhage [16]. Therefore it is important to investigate whether antithrombotic therapy also prevents vascular dysfunction, which is a hallmark of hypertension. Accordingly, we hypothesized that treatment with clopidogrel ameliorates hypertension-associated vascular dysfunction. In addition, we investigated if vascular P2Y_12_ receptor inhibition is involved in the protective effects of clopidogrel on endothelial function. To test our hypothesis, we assessed the effects of clopidogrel treatment on aortic function in control and angiotensin II (Ang II)-treated rats. Initially, we characterized the purinoceptors involved in the vascular actions of 2-methyl-thio-adenosine-diphosphate (2-MeS-ADP), both in control and Ang II-hypertensive rats. Subsequently, we evaluated the effects of clopidogrel-treatment on endothelial-function and wall structure in aortas from control and Ang II-hypertensive rats.

## Methods

### Animals and blood pressure measurement

Ten week-old male Sprague-Dawley rats (230–250 g; Harlan Laboratories, Indianapolis, IN), maintained on a 12∶12-h light-dark cycle with *ad libitum* access to rat chow and water, were used in these studies. All procedures were conducted in accordance with the Guidelines for the Care and Use of Laboratory Animals of the National Institutes of Health and were reviewed and approved by the Institutional Animal Care and Use Committee of the Georgia Regents University (IACUC Approval for Protocol 2009-0226).

Rats were anesthetized with a mixture of ketamine (80 mg.kg^−1^) and xylazine (10 mg.kg^−1^) and osmotic mini pumps (0.5 μl per hour - 14 days - model 2002, Alzet Co., Cupertino, CA) were subcutaneously and dorsally implanted at the base of the neck. Animals were divided into two groups: a control group infused with saline only (control group), and other infused with Ang II (60 ng.min^−1^) for a period of 14 days (Ang II-hypertensive group). Both groups were simultaneously treated either with clopidogrel (Plavix - 10 mg.kg^−1^.day^−1^) or vehicle (peanut butter, 1 g) for 14 days. At day 0 (before experimental procedure) and at day 14, systolic blood pressure (SBP) was measured by tail cuff plethysmography in conscious rats. The efficacy of treatment with clopidogrel was determined by the bleeding time. Briefly, after 14 days of treatment with clopidogrel or vehicle, rats were placed in individual restrainers and the tip of the tail (3 mm) was cut and blood drops were collected on filter paper. The duration of bleeding was recorded.

### Vascular functional studies

After euthanasia, the aorta was rapidly excised and placed in a cold physiological salt solution (PSS, 4°C), containing (mM): NaCl, 130; NaHCO_3_, 14.9; KCl, 4.7; KH_2_PO_4_, 1.18; MgSO_4_·7H_2_O 1.18; CaCl_2_·2H_2_O, 1.56, EDTA, 0.026, glucose 5.5. Aortas were carefully dissected and mounted as ring preparations (≅5 mm in length) in an isometric Mulvany-Halpern myograph (model 610 DMT-USA, Marietta, GA) and recorded by a PowerLab8/SP data acquisition system (ADInstruments Pty Ltd., Colorado Springs, CO). Both dissection and mounting of the vessels were carried out in cold (4°C) PSS. Aortas were stretched to maintain a resting tension of 30 mN, which was determined in preliminary experiments. Accordingly, aortas were submitted to different resting tensions, from 5 to 50 mN, and after a stabilization-period, were stimulated with a contractile agent at each resting tension. The optimal resting tension (30 mN) was chosen based on the largest reproducible contractile responses exhibited by the arteries. Arteries were equilibrated for 45 min in PSS at 37°C, and continuously bubbled with 5% CO_2_ and 95% O_2_. Arterial integrity was assessed first by stimulation of vessels with 120 mM potassium chloride (KCl) and, after washing and a new stabilization time, by contracting the segments with phenylephrine (PE; 10 μM) followed by relaxation with acetylcholine (ACh; 100 μM). Endothelium-dependent relaxation was assessed by measuring the relaxation response to ACh (1 nM to 100 μM) in PE-contracted vessels. ACh, as well as sodium nitroprusside [(SNP) 0.1 nM to 10 μM] responses were also evaluated after incubation with vehicle or with the NO synthase inhibitor N-nitro-*L*-arginine methyl ester (L-NAME 100 μM) plus indomethacin (10 μM), an inhibitor of prostanoid synthesis, for 30 minutes. The response to 2-MeS-ADP (0.1 to 100 μM) was evaluated in arteries on basal tone and after PE-induced (3 μM) contraction, in the presence or absence of L-NAME and indomethacin. To avoid the possibility of tachyphylactic responses, concentration-response curves to 2-MeS-ADP were performed by testing only one concentration of 2-MeS-ADP in each vascular preparation. Therefore, various vascular preparations from one animal were stimulated with only one concentration of 2-MeS-ADP (0.01 to 100 μM), to construct the concentration-response curve. In addition, 2-MeS-ADP-induced responses (both contraction and relaxation) were determined in the presence of selective antagonists for P2Y_1_, P2Y_12_ and P2Y_13_ receptors: MRS-2179, MRS-2395 and MRS-2211, respectively.

### Western blot for detection of vascular P2Y_1_, P2Y_12_ and P2Y_13_ receptors

Proteins (40 μg) extracted from aortas were separated by electrophoresis on a 10% polyacrylamide gel and transferred to a nitrocellulose membrane. Nonspecific binding sites were blocked with 5% skim milk in Tris-buffered saline solution with Tween for 1 hour at 24°C. Membranes were incubated with antibodies overnight at 4°C. Antibodies were as follows: P2Y_1_, P2Y_12_, P2Y_13_ (1∶200; Alomone Labs) and β-actin (1∶1000; Sigma). After incubation with secondary antibodies, signals were revealed with chemiluminescence, visualized by autoradiography, and quantified densitometrically. Results are normalized to β-actin protein and expressed as arbitrary units.

### Vascular structure analysis/morphological analysis

Thoracic aortas were fixed in methacarn solution (60% methanol, 30% chloroform, 10% acetic acid), processed for paraffin embedding in an automated system (SHANDON Citadel tissue processor) and serial sections (5 μm thick) were obtained. Tissue sections were de-waxed with ethanol and stained with hematoxylin-eosin. The aortic media cross-sectional area (CSA) was analyzed with Image-Pro Plus 6.0 (Media Cybernetics, Bethesda, MD, USA), by a single investigator blinded to the experimental groups.

### Data analysis

The results are shown as mean ± SEM where “n” represents the number of rats used in the experiments. Contractions were recorded as changes in the displacement (mN) from baseline and normalized by PE contraction and are represented as percentage of PE-induced contraction. Relaxation is expressed as percent change from the PE contracted levels. Concentration–response curves were fitted using a nonlinear interactive fitting program (Graph Pad Prism 5.0; GraphPad Software Inc., San Diego, CA, U.S.A.). Values of *P*<0.05 were considered a statistically significant difference. Statistical analysis was performed using two-way analysis of variance plus Newman-Keuls post hoc analysis to compare the concentration response curves between all the groups. The other evaluations were performed with one-way analysis of variance plus Newman-Keuls post hoc analysis or Student's t test, where appropriate.

### Chemicals

Acetylcholine chloride, angiotensin II, indomethacin, N-nitro-*L*-arginine methyl ester (L-NAME), 2-(Methylthio) adenosine 5′-trihydrogen diphosphatetrisodium (2-MeS-ADP), MRS-2395 [2,2-Dimethyl-propionic acid 3-(2-chloro-6-methylaminopurin-9-yl)-2-(2,2-dimethyl-propionyloxymethyl)-propyl ester], sodium nitroprusside (SNP) and phenylephrine hydrochloride, were purchased from Sigma Aldrich (St. Louis, MO). Clopidogrel bisulfate (Bristol-Myers Squibb Company, New York, NY, USA) was obtained by crushing Plavix tablets (75 mg). MRS-2179 tetrasodium salt [2′-Deoxy-N6-methyladenosine 3′,5′-bisphosphate tetrasodium salt] and MRS-2211 2 disodium salt ([(2-Chloro-5-nitrophenyl)azo]-5-hydroxy-6-methyl-3-[(phosphonooxy)methyl]-4-pyridinecarboxaldehyde) were purchased from Tocris (Ellisville, MO).

## Results

Systolic blood pressure (mmHg) was not modified in clopidogrel-treated control rats (128±1) vs. vehicle-treated control rats (134±2) or in clopidogrel-treated Ang II-hypertensive rats (190±11) vs. vehicle-treated Ang II-hypertensive rats (199±9).

Bleeding time was 424±31 seconds in vehicle-treated control rats and 470±42 in vehicle-treated Ang II- hypertensive rats. Bleeding time exceeded 1200 seconds in clopidogrel-treated control and clopidogrel-treated Ang II-hypertensive rats.

### 2-MeS-ADP and relaxation

**Figure 1:**
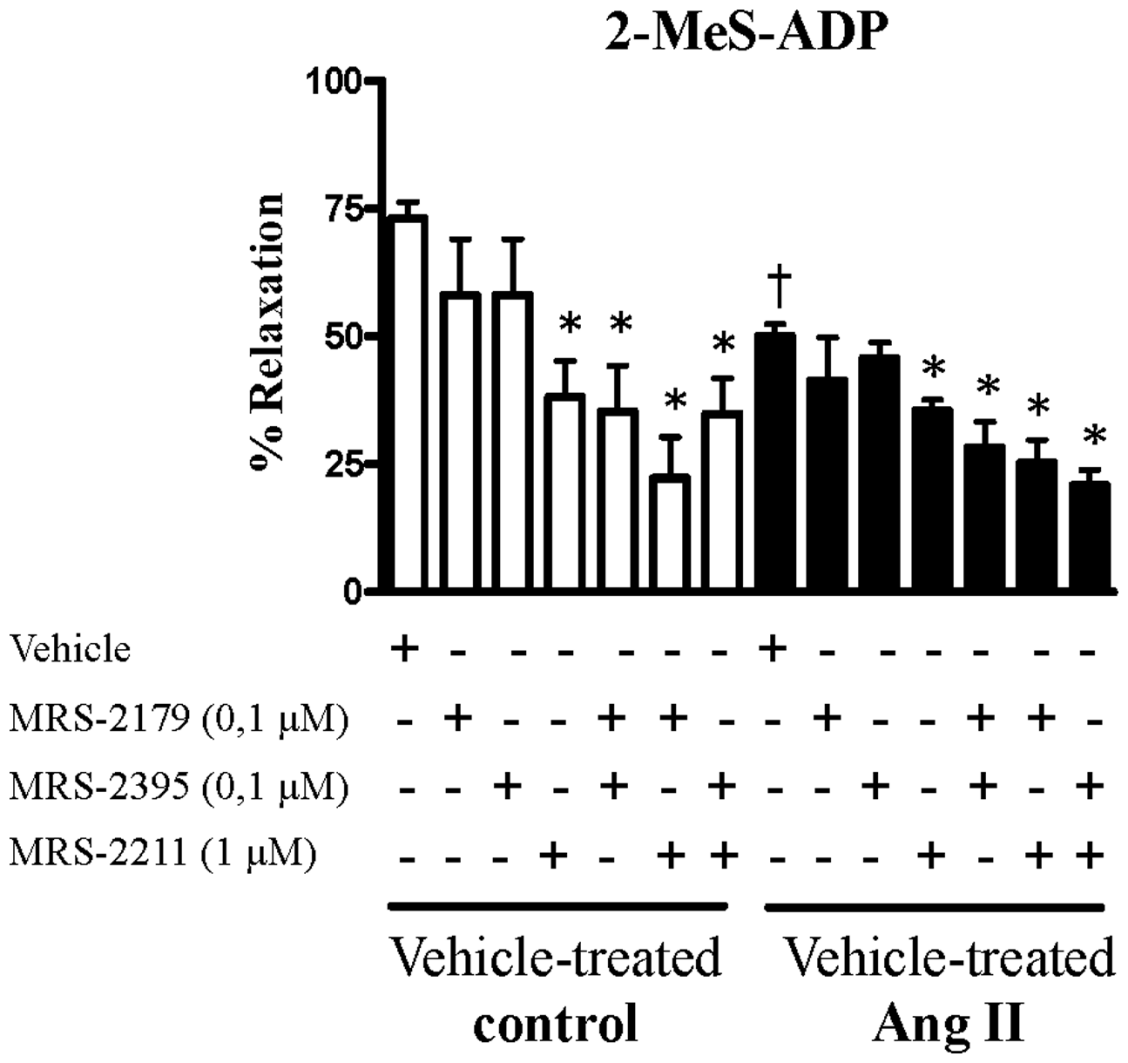
Endothelium-dependent relaxation by 2-MeS-ADP is decreased in aortas from Ang II-hypertensive rats compared to control. Relaxation induced by 2-MeS-ADP (100 μM) in PE-contracted (3 μM) aortas from vehicle-treated control (white bars, n = 5) and vehicle-treated Ang II-hypertensive rats (black bars, n = 6). Experiments were conducted in the presence (+) or absence (−) of MRS-2179 (0.1 μM), a P2Y_1_ receptor antagonist, MRS-2395 (0.1 μM), a P2Y_12_ receptor antagonist and MRS-2211 (1 μM), a P2Y_13_ receptor antagonist. Experimental values of the relaxation induced by 2-MeS-ADP were calculated relative to the maximal changes from the contraction produced by PE in each tissue, which was taken as 100%. Results are presented as mean ± SEM in each experimental group. *P<0.05 vs. respective vehicle-treated control rats. † P<0.05 vs. vehicle-treated Ang II rats.

On resting or basal tension, stimulation of aortic rings with 2-MeS-ADP (0.01–100 μM) did not produce changes in force levels. When 2-MeS-ADP-stimulation was performed in aortas contracted with PE (3 μM), a significant relaxation was observed. Concentration-response curves to 2-MeS-ADP (10 nM to 100 μM) showed that maximum relaxation was obtained with 100 μμM 2-MeS-ADP (65±9%). Endothelium-dependent relaxation was determined in the groups. Aortas from vehicle-treated Ang II-hypertensive rats exhibited decreased endothelium-dependent relaxation to 2-MeS-ADP, compared to vehicle-treated control rats (Figure 1).

2-MeS-ADP is the most potent P2Y_12_ receptor agonist available, but this analogue may also activate P2Y_1_ and P2Y_13_ receptors [22]. Therefore, our next goal was to determine which of these receptors contributes to endothelium-dependent relaxation elicited by 2-MeS-ADP. Aortic rings were incubated with MRS-2179 (0.1 μM), a P2Y_1_ antagonist; MRS-2395 (0.1 μM), a P2Y_12_ antagonist; MRS-2211 (1 μM), a P2Y_13_ antagonist; alone or in combination, for 30 minutes.

Incubation with MRS-2211, but not with MRS-2179 or MRS-2395, reduced the relaxation response induced by 2-MeS-ADP in aortas from vehicle-treated control and vehicle-treated Ang II-hypertensive rats (Figure 1). Simultaneous incubation with MRS-2179 and MRS-2211 further inhibited relaxation responses to 2-MeS-ADP in the vehicle-treated control group. Simultaneous incubation with MRS-2395 and MRS-2211 reduced the relaxation responses to 2-MeS-ADP in a similar pattern compared to MRS-2211 incubation alone. Similar results were observed in aortas from the vehicle-treated Ang II-hypertensive group. These results suggest that in the rat aorta, endothelial P2Y_1_ and P2Y_13_ receptors are major contributors to the endothelium-dependent relaxation elicited by 2-MeS-ADP (Figure 1).

### 2-MeS-ADP and contraction

**Figure 2:**
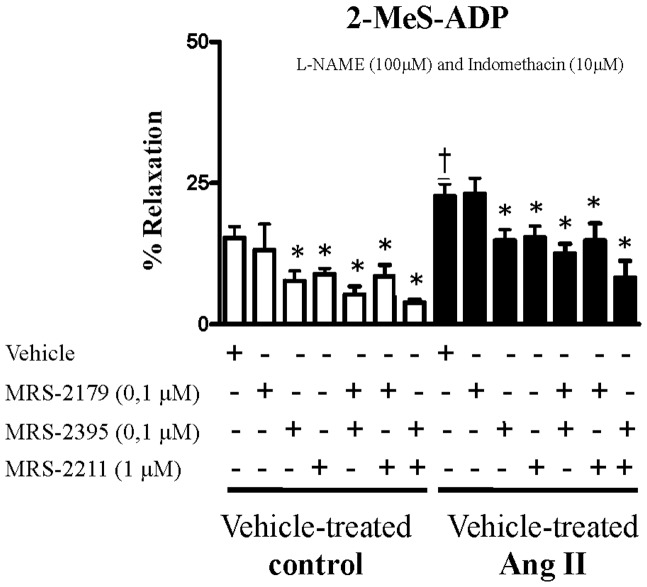
Contraction by 2-MeS-ADP is augmented in aortas from Ang II-hypertensive rats compared control. Contractile response induced by 2-MeS-ADP (100 μM) in aortas from vehicle-treated control (white bars, n = 5) and vehicle-treated Ang II-hypertensive rats (black bars, n = 6) incubated with L-NAME (100 μM) and indomethacin (10 μM) for 30 minutes, to rule out the interference from endogenous release of NO and cyclooxygenase products, respectively. Experiments were conducted in the presence (+) or absence (−) of MRS-2179 (0.1 μM), a P2Y_1_ receptor antagonist, MRS-2395 (0.1 μM), a P2Y_12_ receptor antagonist and MRS-2211 (1 μM), a P2Y_13_ receptor antagonist. Results are presented as mean ± SEM in each experimental group. *P<0.05 vs. respective vehicle-treated control rats. † P<0.05 vs. vehicle-treated Ang II rats.

The next set of experiments was conducted in the presence of L-NAME (100 μM) and indomethacin (10 μM), in order to rule out the influence of products derived from NOS and COX 1 and 2. On resting basal tension, stimulation with 2MeS-ADP (0.01–100 μM) induced vascular contraction, and the maximum contractile response was observed at 100 μM. Next, contractions were determined in the experimental groups. Aortas from vehicle-treated Ang II-hypertensive rats displayed increased contractile responses to 2-MeS-ADP, compared to vehicle-treated control rats (Figure 2).

Incubation with MRS-2179 did not affect 2-MeS-ADP-induced contraction either in the control or Ang II groups. Inhibition with MRS-2395 or with MRS-2211 significantly decreased 2-MeS-ADP-induced contractile responses, both in aortas from vehicle-treated control and vehicle-treated Ang II-hypertensive rats. Simultaneous incubation with MRS-2395 and MRS-2211 further inhibited contractile responses to 2-MeS-ADP in both groups (Figure 2). These results show that endothelium-independent contractile responses elicited by 2-MeS-ADP are mediated by activation of P2Y_12_ and P2Y_13_ receptors.

### Expression of P2Y_1_, P2Y _12_, P2Y _13_ receptors in the rat aorta

**Figure 3:**
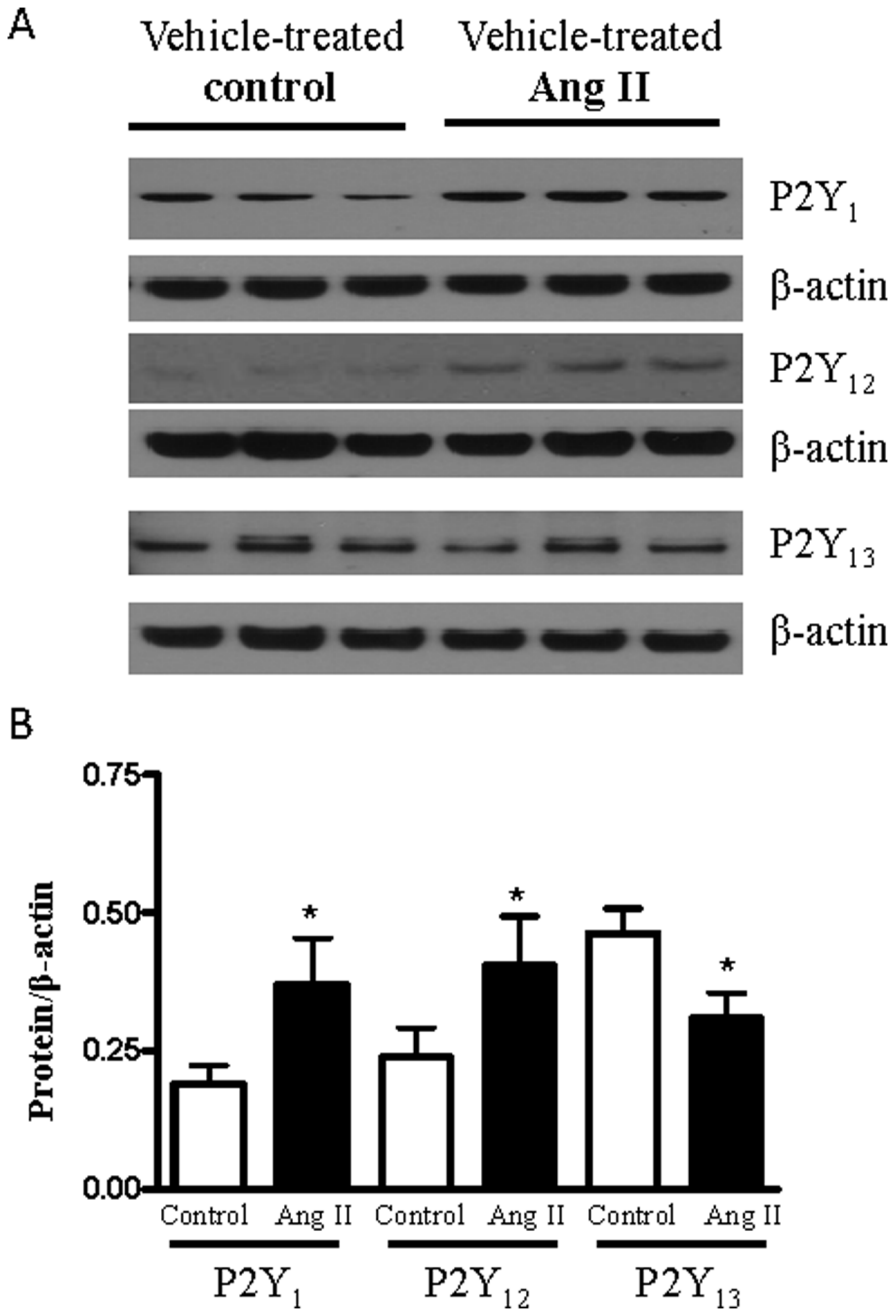
Protein expression of P2Y_1_, P2Y_12_ and P2Y_13_ receptors in aortas from control and Ang II-hypertensive rats. (A) representative images of P2Y receptors expression in rat aortas from vehicle-treated control (n = 4) and vehicle-treated Ang II-hypertensive rats (n = 5). (B) Bar graphs showing the relative vascular expression of P2Y receptors after normalization to β-actin expression. Results are presented as mean ± SEM in each experimental group. * P<0.05 vs. respective vehicle-treated control rats.

Protein expression of P2Y_1_, P2Y_12_ and P2Y_13_ receptors was measured in aortas from vehicle-treated control and vehicle-treated Ang II-hypertensive rats. P2Y_1_ and P2Y_12_ protein expression was increased, whereas P2Y_13_ receptor expression was reduced, in aorta from vehicle-treated Ang II-hypertensive rats compared to the vehicle-treated control group (Figure 3).

### Clopidogrel-treatment, 2-MeS-ADP and endothelial function

Rats from control and Ang II-hypertensive groups were treated with vehicle or clopidogrel, for 14 days, to evaluate 2-MeS-ADP responses, endothelial function and vascular remodeling.

**Figure 4:**
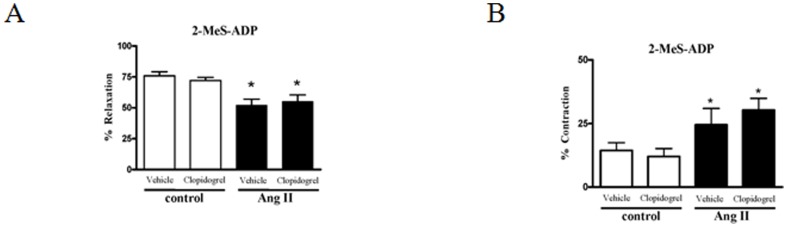
2-MeS-ADP induced-responses are not altered by treatment with clopidogrel. (**A**) Relaxation induced by 2-MeS-ADP (100 μM) in PE-contracted (3 μM) aortas. (**B**) Contractile response induced by 2-MeS-ADP (100 μM) in aortas incubated with L-NAME (100 μM) and indomethacin (10 μM) for 30 minutes. Aortas from control (white bars, n = 5) and Ang II-hypertensive rats (black bars, n = 5) treated with vehicle or with clopidogrel. Experimental values of the relaxation induced by 2-MeS-ADP were calculated relative to the maximal changes from the contraction produced by PE in each tissue, which was taken as 100%. Results are presented as mean ± SEM in each experimental group. *P<0.05 vs. respective control group (vehicle-treated control or clopidogrel-treated control).

The relaxation-responses induced by 2-MeS-ADP were reduced in aortas from vehicle-treated Ang II-hypertensive rats and this reduction was not affected by clopidogrel (Figure 4A). The contractile-responses to 2-MeS-ADP were augmented in aortas from vehicle-treated Ang II-hypertensive and it was also unaffected by clopidogrel-treatment (Figure 4B).

Endothelium-dependent relaxation was assessed based on the responses to ACh, whereas endothelium-independent relaxation was assessed by determining responses to SNP.

**Figure 5:**
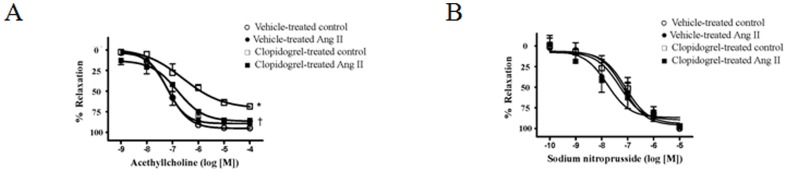
Clopidogrel improves ACh-induced relaxation in aortas from Ang II-hypertensive rats. Concentration-response curves to (**A**) ACh (without NOS and COX inhibitors incubation) or (**B**) SNP in the presence of L-NAME (100 μM) plus indomethacin (10 μM) in arteries from (○) vehicle–treated control (n = 5); (•) clopidogrel-treated control (n = 5); (□) vehicle-treated Ang II-hypertensive (n = 6); and (▪) clopidogrel-treated Ang II hypertensive-rats (n = 6). Experimental values of the relaxation induced by ACh were calculated relative to the maximal changes from the contraction produced by PE in each tissue, which was taken as 100% (% of relaxation). Results are presented as mean ± SEM in each experimental group. *, P<0.05 vs. vehicle-treated control rats; †, P<0.05 vs. vehicle-treated Ang II rats.

Aortas from vehicle-treated Ang II-hypertensive rats exhibited impaired relaxation to ACh, which was significantly improved in aorta from clopidogrel-treated Ang II-hypertensive rats. No differences were observed in ACh responses between aortas from vehicle- and clopidogrel-treated control rats (Figure 5A). No differences in SNP-induced relaxation were observed for any of the groups (Figure 5B).

### Vascular structural analysis

**Figure 6:**
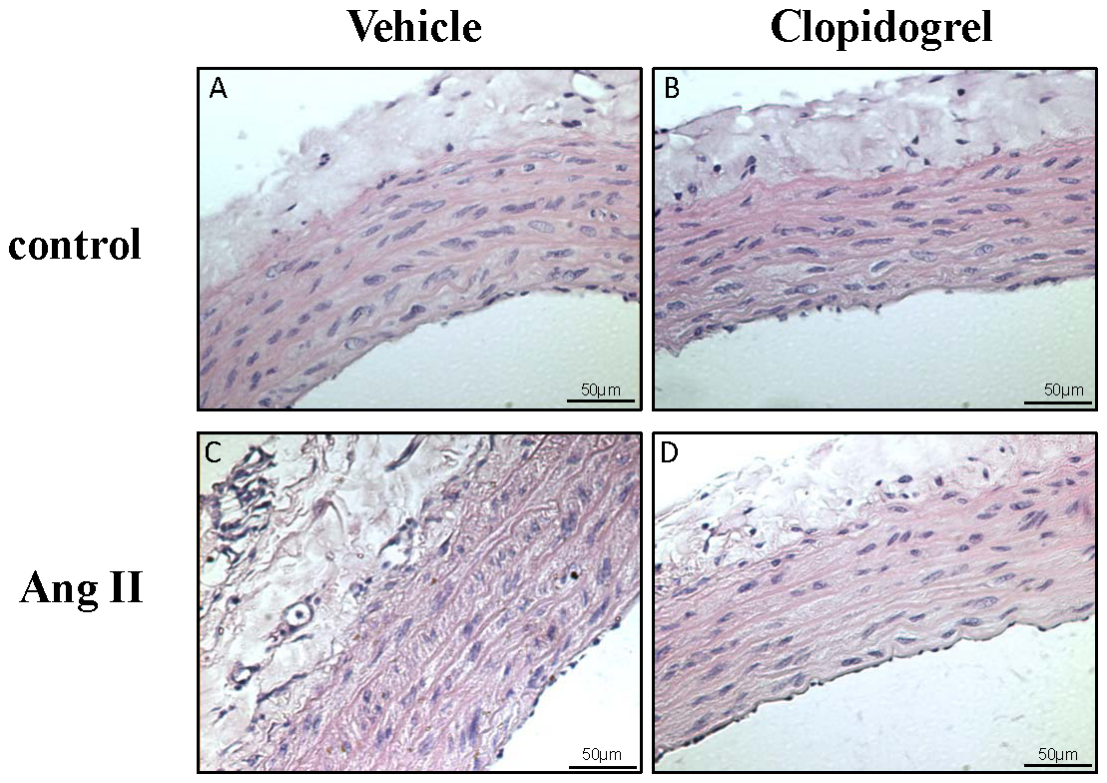
Clopidogrel improves vascular remodeling in aortas from Ang II-treated rats. Representative sections of aortas from (A) vehicle-treated control, (B) clopidogrel-treated control, (C) vehicle-treated Ang II-hypertensive and (D) clopidogrel-treated Ang II-hypertensive rats. Slices were stained with hematoxilin-eosin. n = 4 in each group.

Ang II infusion resulted in enlargement of aortic media thickness and the media-lumen ratio. Treatment with clopidogrel partially prevented media thickening and increased media-lumen ratio in Ang II-hypertensive rats. A tendency to decrease internal lumen diameter was observed in aortas from clopidogrel-treated Ang II-hypertensive rats. Treatment with this anti-platelet agent had no effects on the vascular structural parameters in control rats (Figure 6 - Table 1).

**Table 1:** Effects of clopidogrel-treatment on media thickness, lumen and media-lumen ratio in control and Ang II-hypertensive rats, treated with vehicle or with clopidogrel. n = 4 in each group.

	Vehicle-treated control	Clopidogrel-treated control	Vehicle-treated Ang II	Clopidogrel-treated Ang II
Media thickness (μm)	87.35±2.5	90.8±2.5	135±4.1*	107.89±6.8†
Lumen (μm)	1226±58	1204±24	1110±17.22	1201±49
Media-Lumen ratio (%)	7.14±0.1	7.53±0.1	12.21±0.3*	9.01±0.2†

* p<0.01 vs. vehicle-treated control;

† p<0.01 vs. vehicle-treated Ang II.

## Discussion

The results of the current study demonstrate that clopidogrel treatment prevented aortic dysfunction and remodeling in hypertensive rats. Intravascular pressure is one of the mechanisms that contribute to vascular dysfunction in hypertension and one may argue that the improvements observed in hypertensive rats treated with clopidogrel may be due to a reduction in blood pressure. However, there were no differences in blood pressure among the groups at the end of clopidogrel treatment. Clopidogrel had no detectable effects on systolic blood pressure in the control rats and Ang II-infused rats were similarly hypertensive after 14 days with or without clopidogrel treatment. Similarly, renal injury in Ang-II hypertensive rats was ameliorated by treatment with clopidogrel independently of a reduction in arterial pressure [17].

Vascular reactivity experiments were performed to evaluate possible mechanisms by which clopidogrel may improve aortic function during hypertension. The aorta was chosen because its function may be just as important in hypertension as increased peripheral vascular resistance [18], when one considers the relative impact of the sustained and pulsatile components of the blood pressure profile across the cardiac cycle. Mean arterial pressure is relatively constant along the arterial tree, but pulse pressure increases from the more central to the peripheral arteries. Accordingly, large arteries cannot be considered just passive conduits in hypertension because they display clearly measurable functional changes in response to the mechanical forces delivered by significantly elevated blood pressure [18].

In a variety of vessels and species, ADP-evoked relaxations are mediated mainly by P2Y_1_ receptors [19] whereas the role of P2Y_12_ and P2Y_13_ receptors requires further study. The P2Y_1_ receptor antagonist MRS-2179 strongly inhibits ADP-induced platelet aggregation both *in vitro* and *ex vivo* [20]. MRS-2179 is a pure competitive antagonist of P2Y_1_ receptors in transfected cell lines, with a pA_2_ value of 6.99 [21]. The P2Y_12_ receptor antagonist MRS-2395 inhibits platelet aggregation without effects on P2Y_1_ receptors [22]. The P2Y_13_ receptor antagonist MRS-2211 acts as a competitive antagonist and has a 20-fold selectivity for the P2Y_13_ receptors, compared to P2Y_1_ and P2Y_12_ receptors [22], [23].

Our data show that 2-MeS-ADP induced endothelium-dependent relaxation of pre-contracted aortas occurs primarily through P2Y_1_ and P2Y_13_ receptors. Additionally, 2-MeS-ADP-induced relaxation is blunted in aortas from Ang II- hypertensive rats, compared to control. Dol-Gleizes and colleagues showed that P2Y_1_ receptors were involved in 2-MeS-ADP evoked relaxation responses in the aorta. Furthermore, they showed that clopidogrel did not modify the vasorelaxant effects of 2-MeS-ADP and they concluded that clopidogrel has no pharmacological target other than platelets [6]. Consistent with the work of Dol-Gleizes et al, we did not find a role for P2Y_12_ receptors in the relaxation response to ADP in aorta. Notably, P2Y_12_ receptors were identified in brain capillary endothelial cells [24] and in smooth muscle cells from human blood vessels, where their activation induces contraction [25]. So the potential roles of P2Y_12_ receptors in the regulation of cardiovascular function continue to diversify.

The reduced relaxation response to 2-MeS-ADP observed in aortas from hypertensive rats may be explained, in part, by differential expression of purinoceptors between the normotensive and hypertensive groups. Interestingly, P2Y_13_ receptor expression was lower in aortas from Ang II- hypertensive rats, compared to control. Our pharmacological data endorse the functional presence of P2Y_13_ in the endothelial cells, but warrant confirmation using molecular techniques. Endothelial cell expression of P2Y_1_ and P2Y_12_ receptors has been previously reported [26], [27], but it seems that P2Y_12_ receptors do not play a direct role in ADP-induced aortic relaxation. However, improvement of endothelial function due to P2Y_12_ receptor blockade may occur by different mechanisms, which may not depend on this receptor in the vasculature.

The best known effect of ADP in the vasculature is vasodilation [25], [28], but we have shown that ADP can cause vasoconstriction through the activation of P2Y_12_ and P2Y_13_ receptors in aortas incubated with L-NAME and indomethacin. P2Y_12_ and P2Y_13_ receptors are expressed in the VSMC [27], [29]. ADP stimulates contraction of human blood vessels through P2Y_12_ receptor activation with P2Y_1_ and P2Y_13_ receptors yielding smaller contributions to this response [25]. The ADP-induced contractile response was greater in Ang II-hypertensive rats possibly reflecting the greater expression of P2Y_12_ receptors, with significant endothelial dysfunction in the hypertensive rats.

Aortas from Ang II-hypertensive rats treated with clopidogrel displayed greater ACh-induced relaxation, compared to aortas from vehicle-treated hypertensive rats, indicating improvement in endothelial cell function, since endothelium-independent relaxation was similar in all groups. It is a consensus that the endothelium is an important regulator of vascular tone and platelet aggregation and adhesion. Furthermore, endothelial dysfunction is involved in the pathophysiology of several diseases, and is an earlier predictor of atherosclerosis, a disease where platelet activation importantly contributes to endothelial dysfunction [10]. Accordingly, clopidogrel reportedly improves endothelial function in humans [14], [30], [31], and in experimental models of diseases [15], [32], [33], [34]. There may be major additional benefits conferred by clopidogrel treatment particularly for patients with atherosclerotic risk and impaired arterial function [35]. Notably, even a single dose of clopidogrel improved vascular function in some patients with coronary artery disease, demonstrated by a significantly improved Endoscore, which provides an index of endothelial function, optimal platelet inhibition and reduced circulating levels of endothelial microparticles [36].

Modulation of vascular reactivity by activated platelets depends on a balance of vasodilator activity, mediated primarily by adenine nucleotides, and vasoconstrictor activity, mediated by serotonin and thromboxane. Normal activated human platelets release almost 20-fold more ADP than serotonin and approximately 600-fold more adenine nucleotides than thromboxane [37]. Thus, the predominant response to activation of normal human platelets is vasodilatation. During hypertension, inflammation of the vascular wall is associated with endothelial dysfunction [38], in part, because under such conditions, release of cyclooxygenase (COX)-derived vasoconstrictors, including thromboxane, becomes predominant [39]. Intraluminal activation of normal platelets produced pronounced dilatation of PE-contracted carotid arteries, but vasodilator responses were greatly impaired in hypercholesterolemic patients [40], suggesting that in some conditions, the platelets assume a role to increase production of vasoconstrictor substances, leading to vascular dysfunction.

Ang II participates in the pathogenesis of endothelial dysfunction and vascular remodeling in hypertension [41]. This occurs through several mechanisms, including release of vasoactive mediators by activated platelets, low-grade inflammation and disturbances in nitric oxide bioavailability [42]. Ang II stimulates platelet activation and increases secretion of plasminogen activator inhibitor type I from vascular endothelial cells [43]. These effects of Ang II could be blunted by prevention of platelet activation, because Clopidogrel treatment suppresses platelet activation and platelet-leukocyte aggregate formation. It also leads to reductions in serum levels of CD40 ligand, C-reactive protein and P-selectin. These beneficial effects of clopidogrel on inflammatory markers have been demonstrated in acute ischemic stroke patients, in patients with peripheral arterial disease and across a spectrum of atherothrombotic diseases [44]. Thus the ability of clopidogrel to improve endothelial function in our study supports the idea that clopidogrel may have important beneficial effects that go beyond simple P2Y_12_ receptor inhibition and may provide significant clinical benefit to aortic and large artery function in hypertension.

Another beneficial finding from this study is that clopidogrel-treatment was effective in preventing vascular wall remodeling in hypertensive rats. This is particularly important given that arteries undergo significant remodeling and hence, become “stiffer” as a consequence of elevated arterial pressure. Aortas from vehicle-treated Ang II-hypertensive rats exhibited increased media thickening and increased media-lumen ratio, indicating that this vessel undergoes a hypertrophic inward remodeling. This increase in aortic stiffness coincides with reduced vasodilatory ability particularly under higher pressures. Increased vascular stiffness propagates accelerated transmission of the pulse pressure wave further along the arterial vascular tree and can have a severe impact on resistance vessel integrity and function [45]. Considering that the aorta is the largest vessel in the arterial tree and that during hypertension both systolic and diastolic pressures are dramatically changed, it is important to better understand the behavior of this vascular bed and identify the positive aspects related to anti-platelet therapy during hypertension [18].

Ang II has pleiotropic actions in multiple systems. In the vasculature, it induces contraction and relaxation, cell growth, migration and differentiation and is pro-fibrotic, pro-oxidant and pro-inflammatory. Subsequent alterations of vascular smooth muscle cell growth, migration, differentiation, production of extracellular matrix proteins and inflammation are then responsible for the resulting vascular remodeling, a hallmark of hypertension. Of the numerous factors influencing remodeling in hypertension, Ang II appears to be one of the most important [46]. Our data show that clopidogrel treatment prevents vascular hypertrophy, decreasing the vascular remodeling induced by Ang II. Although anti-platelet treatment may be effective in preventing vascular remodeling, more studies are necessary to address the mechanisms involved in this process.

## Conclusion

In conclusion, clopidogrel has beneficial effects on aortic structure and function. The mechanism by which this anti-platelet therapy improves vascular function remains to be further evaluated, but it does not seem to be directly related to blockade of P2Y_12_ receptors in the vascular wall.
